# A series of NSAID-induced anaphylactic response to immunotherapy and a proposal to include NSAIDS avoidance in future immunotherapy guidelines

**DOI:** 10.1186/1710-1492-10-S1-A15

**Published:** 2014-03-03

**Authors:** Jennifer YF Chen, Jason K Lee

**Affiliations:** 1 Faculty of Medicine and Dentistry, University of Alberta, Edmonton, AB, Canada; 2 Division of Clinical Immunology and Allergy, University of Toronto, Toronto, ON, Canada; 3 St. Michael’s Hospital, Toronto, ON, Canada

## 

A review of anaphylaxis in a clinic over three years was performed. A co-factor for anaphylaxis stood out as a commonality to all reactions. All five patients had a non steroidal anti-inflammatory drug (NSAID) 24 hours prior to immunotherapy.

Among symptoms, urticaria was the commonest; others included cough, angioedema, dyspnea, nausea, vomiting, hypotension and tachycardia. The symptoms appeared within 5 minutes to two hours post-injection. All reactions resolved after few hours. In all cases, NSAID use pre-injection was the only common factor. Two patients also had moderate exercise post-injection, but previous immunotherapy was without incident. All but one patient were administered epinephrine in clinic and recovered without significant morbidity, some were also given cetirizine as adjunct treatment and all were observed until symptoms resolved. While two patients stopped immunotherapy, three patients continued without incident and are now on maintenance dose. The one patient who did not receive epinephrine presented to a walk in clinic for her reaction and received antihistamine treatment alone.

NSAID use, although overlooked in the literature, is a common cofactor in anaphylaxis in response to immunotherapy. At Torontoallergists™, a clinic with three allergists in practice, 5 such cases among approximately 3 600 injections in the last three years were noted after extensive chart review. Therefore an NSAID was associated with anaphylaxis in 0.0014% of the immunotherapy injections, whereas two cases of anaphylaxis involved NSAID and exercise involvement. No cases implicating other risk factors or co-factors for anaphylaxis during immunotherapy, such as dosage errors, or injection during asthma exacerbation were present [1].

It is speculated that aspirin and other NSAIDS lower the threshold for anaphylaxis after allergen injections through their COX-inhibiting mechanism of action [2]. The COX pathway synthesizes, from arachidonic acids, prostaglandin D2 and E2, are repressors of inflammatory mediator release from basophils and mast cells [3]. Therefore, NSAIDS increases the likelihood of anaphylaxis after immunotherapy by suppression of prostaglandin D2 and E2, which would normally inhibit histamine release. Supporting this mechanism is Marone *et al*’s study where higher concentration of NSAIDS and more inhibition of COX activity correlated with higher release of histamine [2].

However, in spite of the data about NSAID acting as a co-factor for anaphylaxis, NSAID avoidance around immunotherapy cannot be found in practice parameters from the AAAAI, ACAAI, and CSACI [4,5].

Therefore, as a patient safety precaution, we highly encourage NSAIDS avoidance to be included for future immunotherapy practice guidelines. We welcome other centers to corroborate.

## References

[B1] Borchers AT Keen CL Gershwin ME Fatalities following allergen immunotherapy Clin Rev Allergy Immunol 2004 10 147 58 15576898 10.1385/CRIAI:27:2:147

[B2] Marone G Kagey-Sobotka A Lichtenstein LM Effects of arachidonic acid and its metabolites on antigen-induced histamine release from human basophils in vitro J Immunol 1979 10 1669 77 90097

[B3] Hogaboam CM Bissonnette EY Chin BC Befus AD Wallace JL Prostaglandins inhibit inflammatory mediator release from rat mast cells Gastroenterology 1993 10 122 9 7678236 10.1016/0016-5085(93)90843-2

[B4] Cox L Nelson H Lockey R Calabria C Chacko T Finegold I Nelson M Weber R Bernstein DI Blessing-Moore J Allergen immunotherapy: a practice parameter third update J Allergy Clin Immunol 2011 10 S1 55 21122901 10.1016/j.jaci.2010.09.034

[B5] Leith E Bowen T Butchey J Fischer D Kim H Moote B Small P Stark D Waserman S Consensus Guidelines on Practical Issues of Immunotherapy-Canadian Society of Allergy and Clinical Immunology (CSACI) Allergy Asthma Clin Immunol 2006 10 47 61 20525157 10.1186/1710-1492-2-2-47 PMC2876183

